# Uptake and 4-week outcomes of an ‘opt-out’ smoking cessation referral strategy in a London-based lung cancer screening setting

**DOI:** 10.1136/bmjresp-2024-002337

**Published:** 2025-02-06

**Authors:** Amyn Bhamani, Evangelos Katsampouris, Fanta Bojang, Priyam Verghese, Andrew Creamer, Ruth Prendecki, Chuen R Khaw, Jennifer L Dickson, Carolyn Horst, Sophie Tisi, Helen Hall, John McCabe, Kylie Gyertson, Anne-Marie Hacker, Laura Farrelly, Neal Navani, Allan Hackshaw, The SUMMIT Consortium, Sam M Janes, Samuel M Janes, Samantha L Quaife

**Affiliations:** 1 Lungs for Living Research Centre, UCL Respiratory, University College London, London, UK; 2 Wolfson Institute of Population Health, Queen Mary University of London, London, UK; 3 University College London Hospitals NHS Foundation Trust, London, UK; 4 Cancer Research UK and UCL Cancer Trials Centre, London, UK

**Keywords:** Lung Cancer, Tobacco and the lung

## Abstract

**Introduction:**

Lung cancer screening (LCS) enables the delivery of smoking cessation interventions to a population experiencing long-term tobacco dependence, but the optimal delivery method remains unclear. Here, we report uptake and short-term outcomes of an ‘opt-out’ smoking cessation referral strategy in an LCS cohort.

**Methods:**

Individuals currently smoking tobacco who attended a face-to-face lung health check in the SUMMIT study (NCT03934866) were offered very brief advice on smoking cessation and where possible, an ‘opt-out’ referral to their local stop smoking service (SSS). Aggregate data on referral outcomes were obtained from each SSS individually.

**Results:**

33.7% (n=2090/6203) of individuals currently smoking tobacco consented to a practitioner-made ‘opt-out’ smoking cessation referral. 42.7% (n=893/2090) of these individuals resided in boroughs where SSS were not present or required self-referral. Males (adjusted OR (aOR) 1.16), younger individuals (55–59: aOR 1.70, 60–64: aOR 1.71 and 65–69: aOR 1.78) and those of ethnic minority backgrounds (Asian: aOR 1.31, Black: aOR 1.71 and Mixed: aOR 1.72) were more likely to consent, while individuals from the most deprived socioeconomic quintile were less likely to do so (aOR 0.65).

High level of motivation to quit within a defined time frame (aOR 1.92), previous quit attempts in the past 12 months (1–4: aOR 1.65 and ≥5: aOR 1.54) and time to first cigarette of ≤60 min (<5: aOR 2.07, 6–30: aOR 1.55 and 31–60: aOR 1.56) were measures of tobacco dependence associated with a higher likelihood of providing consent.

Outcomes were available for 742 referrals. An appointment with the service was accepted by 47.3% (n=351/742) of individuals, following which 65.5% (n=230/351) set a quit date. The 4-week quit rate among those setting a quit date and all individuals referred was 57.4% (n=132/230) and 17.8% (n=132/742), respectively.

**Conclusion:**

A proactive, ‘opt-out’ smoking cessation referral strategy for individuals currently smoking tobacco who interact with an LCS programme may be beneficial.

**WHAT IS ALREADY KNOWN ON THIS TOPIC**

- Participation in lung cancer screening (LCS) increases motivation to stop smoking and enables the delivery of smoking cessation interventions to a population experiencing long-term tobacco dependence.

**WHAT THIS STUDY ADDS**

- This study reports stop smoking service (SSS) engagement outcomes of a practitioner-made, ‘opt-out’ smoking cessation referral strategy in a large LCS cohort. Our results suggest that adopting a proactive approach to smoking cessation referrals in this setting is both feasible and beneficial.

**HOW THIS STUDY MIGHT AFFECT RESEARCH, PRACTICE OR POLICY**

- Our findings suggest that a clinician-made ‘opt-out’ referral strategy is effective and should be considered as a minimum standard of care for LCS programmes. However, referral pathways and the provision of SSS require further optimisation in order to ensure that the full benefit of this strategy in the context of LCS can be realised.

## Introduction

 Lung cancer is the leading cause of cancer-related deaths in the United Kingdom (UK), accounting for nearly 35 000 deaths annually.^1^ Low-dose CT (LDCT) screening reduces lung cancer-related mortality among high-risk individuals by enabling diagnosis at an earlier stage^2 3^ and, in 2022, targeted screening for this condition was recommended by the UK National Screening Committee.^4^

Eligibility for lung cancer screening (LCS) is based on lung cancer risk, determined primarily by age and a significant and recent tobacco smoking history. Indeed, an estimated 86% of lung cancer deaths in the UK are caused by tobacco smoking,^5^ while up to 55% of participants in LCS cohorts are reported to be individuals who currently smoke at the time of enrolment.^2^

Participation in LCS can increase motivation to stop smoking^6^ and is associated with increased rates of smoking cessation compared with the general population.^7^ LCS visits, therefore, provide a valuable opportunity for smoking cessation interventions to be delivered to a population with long-term tobacco dependence. Despite this, it remains unclear how such interventions are best provided in this setting. In 2017, a multistakeholder committee convened by the American Thoracic Society noted that the optimal method and timing of smoking cessation counselling and pharmacotherapy in the context of LCS remain unclear. It was recommended that *‘a national research agenda should include strategies for implementing tobacco dependence treatment within the LCS setting’.*^8^ In the UK, a recent report from Action on Smoking and Health also highlighted the absence of a clear-cut solution for delivering effective smoking cessation support in an LCS setting. The report emphasised the importance of drawing insights from real-world experiences gained through UK screening pilot programmes to help inform the delivery of smoking cessation interventions in this context.^9^

Evidence suggests that simply advising individuals to quit is inadequate. Instead, proactively offering smoking cessation assistance to all individuals who smoke results in more quit attempts compared with giving advice alone,^10^ with potential benefits being demonstrated across a range of healthcare settings. For instance, in Canada, the implementation of a proactive, ‘opt-out’ smoking cessation referral strategy significantly increased the acceptance of smoking cessation support among patients with cancer,^11^ while in the UK, switching to an ‘opt-out’ referral strategy doubled the number of pregnant smokers reporting smoking cessation after 4 weeks^12^ compared with the previous ‘opt-in’ approach.

In an LCS setting, the National Lung Screening Trial reported that assisting and arranging follow-up smoking cessation support increased the odds of successfully quitting by 40% and 46%, respectively.^13^ In addition, delivering ‘intensive’ telephone-based smoking cessation support has been shown to increase short-term quit rates compared with ‘minimal’ intervention, although no impact on longer term abstinence rates has been demonstrated.^14^ Nevertheless, these data suggest that adopting a proactive ’opt-out’ approach to the delivery of smoking cessation interventions may also be beneficial in the context of LCS. In support, the Yorkshire Enhanced Stop Smoking Study (YESS) found that over 88% of individuals attending LCS study visits accepted an immediate in-person consultation with a co-located smoking cessation advisor during their LCS appointment.^15^ In addition, half of those screened in the Lung Screen Uptake Trial (LSUT) accepted a practitioner-made ‘opt-out’ referral to local stop smoking services (SSSs) during an LCS visit.^16^ However, data on subsequent engagement and quit attempts with services were not reported.

An ‘opt-out’ referral policy (i.e., practitioner-made referral) to a local SSS was implemented for the SUMMIT study (NCT03934866). Here, for the first time in the context of a UK-based LCS cohort, we assess the outcomes of these referrals on service engagement and quit attempts.

## Methods

### Participant recruitment and data collection

The SUMMIT study is a prospective observational cohort study, which aims to assess the implementation of LDCT screening for lung cancer in a high-risk population in North Central and East London and validate a multicancer early detection blood test (NCT03934866).

Individuals invited to undergo eligibility assessment for screening via participation in the study were 55–77-year-old current or former smokers identified from primary care records. Following completion of an initial telephone screener, eligible individuals attended a face-to-face lung health check (LHC) appointment, where questions were asked to assess lung cancer risk. Individuals meeting either the 2013 United States Preventive Services Task Force LDCT screening criteria^17^ or having a ≥1.3% 6-year lung cancer risk based on the Prostate, Lung, Colorectal and Ovarian (PLCOM2012) model^18^ were invited to participate in the study. Participants were asked to provide written consent to all aspects of the study protocol, including LDCT and blood draw.

Data obtained from individuals attending a baseline LHC appointment included a self-reported assessment of respiratory symptoms, ethnicity, highest level of education achieved and medical and smoking histories. Age and gender were obtained from primary care records and residential postcodes were converted into index of multiple deprivation (IMD) ranks to provide an area-level measure of socioeconomic deprivation.

### Assessment of smoking history and smoking cessation referrals

An assessment of smoking history, including duration and average consumption, and current smoking status, categorised as individuals who currently smoke or formerly smoked, was included in the baseline LHC appointment. Questions explored both the use of cigarettes and ‘non-cigarette’ tobacco-based products, including cigars, cigarillos and pipes.

Cigarette pack year values were calculated by the study’s database using the number of years smoked and average consumption. In keeping with previous research, only plausible values for daily consumption (1–80 cigarettes per day or equivalent in grams of tobacco per week) and started smoking age (≥5 years old) were included in the analysis.^19^

All individuals who reported smoking cigarettes on most days of the week and those smoking cigars, cigarillos and pipes at least once per week at the time of the LHC were classified as current tobacco smokers. These individuals were offered very brief advice (VBA; accredited by the National Centre for Smoking Cessation and Training)^20^ on smoking cessation by the research nurse or clinical trials practitioner conducting the appointment regardless of their eligibility for screening and study participation. They were advised that where possible, it was routine policy to refer individuals who smoke to their local SSS for support with managing their smoking. A smoking cessation referral was made on behalf of individuals who consented and were residents in boroughs where local SSSs were present and accepted secondary care referrals. As per protocol, referrals were sent individually via electronic mail to SSS by the administration team within 2 working days of LHC attendance.

Individuals residing in boroughs with no local SSS or where the local service only accepted self-referrals were given this information by staff members conducting appointments. These individuals were provided smoking cessation support cards, which included contact details for local SSS (where available) and the Stop Smoking London Advice Line.

### Participant questionnaire and measures of tobacco dependence

All study participants were asked to complete an electronic questionnaire, which collected further information on level of tobacco dependence (among participants who currently smoked), general health and lifestyle and family history of cancer.

Measures of tobacco dependence included an assessment of motivation to quit using the motivation to stop scale (MTSS),^21^ confidence in being able to stop among those wanting to do so, the number of serious quit attempts made in the past 12 months and time to first cigarette (TTFC) after waking.

The seven possible response options in the MTSS were dichotomised to enable comparison between high motivation to stop in a defined time frame (‘I REALLY want to stop smoking and intend to in the next 3 months’ and ‘I REALLY want to stop smoking and intend to in the next month’) and all other possible responses (‘I do not want to stop smoking’, ‘I think I should stop smoking but do not really want to’, ‘I want to stop smoking but have not thought about when’, ‘I REALLY want to stop smoking but I do not know when I will’ and ‘I want to stop smoking and hope to soon’).^22^ Quit attempts in the past year (0, 1–4 attempts and ≥5 attempts) and TTFC (≤5 min, 6–30 min, 31–60 min and >60 min^23^) were analysed categorically.

### Referral outcomes

SSSs were contacted individually for aggregate referral outcome data. This included the number of referrals and the number of individuals who accepted the service, defined as those who agreed to be booked into a clinic appointment after being contacted. Information on the number who declined support when contacted and those whom the service could not contact following a referral from the study was also collected.

The current standard for the assessment of quit success rates is carbon monoxide (CO)—verified 4-week quit rate—although self-reported 4-week quit rates are also widely used in practice. As most people who smoke relapse within the first couple of days of a serious quit attempt and prognosis for permanent cessation improves fivefold after the first 4 weeks, this time frame is expected to provide a reliable indicator of long-term abstinence.^24^ Therefore, outcome data for those attending a first appointment after accepting the service included the number of individuals that set a quit date, the number of successful 4-week quits (including both CO validated and unvalidated, ie, self-reported only, quits), the number of non-quits and the number lost to follow-up. UK National Health Service (NHS) Digital data definitions were used to define each of these groups.^25^

### Study outcome measures and statistical analysis

The primary analysis assessed referral outcomes, including information on the proportion of individuals who accepted the service following a referral from the study, and the number of successful 4-week quits. Secondary analyses assessed demographic characteristics of those currently smoking tobacco and factors associated with a higher likelihood of consenting to a smoking cessation referral.

All individuals attending a baseline LHC between 8 April 2019 and 31 January 2020 were included in the analysis. This cut-off date was selected considering the impact of the global SARS-CoV-2 pandemic, which resulted in significant disruptions to national healthcare provision, including smoking cessation services, from March 2020.

Descriptive frequencies were calculated for all outcome measures. Multivariate binary logistic regression analysis was used to explore the association of smoking cessation referral acceptance with individual characteristics. The regression model included demographic characteristics (age, gender, smoking history, ethnicity and IMD quintile), measures of tobacco dependence (motivation to stop smoking, previous quit attempts and TTFC) and a known history of chronic obstructive pulmonary disease (COPD). Statistical analysis was carried out using IBM SPSS statistics (version 28.0.0). A p value of<0.05 was considered statistically significant.

### Patient and public involvement (PPI)

A PPI group was regularly involved in the setup and design of the SUMMIT study. The group reviewed the study protocol and supporting documents, including invitation materials, participant information sheet, consent form and results letters on several occasions.

## Results

### Sample characteristics

**Figure 1:**
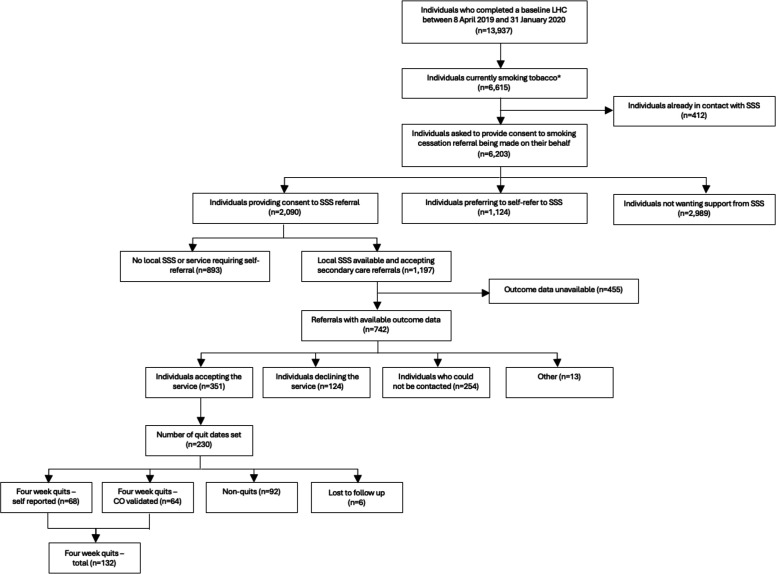
Flow diagram showing journey from attendance at baseline lung health check appointment through to interaction with local stop smoking services. *Includes both cigarette and non-cigarette tobacco smokers. CO, carbon monoxide; SSS, stop smoking service.

13 937 individuals completed a baseline LHC between 8 April 2019 and 31 January 2020. 46.9% (n=6537/13 937) currently smoked cigarettes. A further 0.6% (n=78/13 937) of individuals smoked cigarillos, cigars or a pipe, but not cigarettes regularly. Overall, 47.5% (n=6615/13 937) were classified as currently smoking tobacco (figure 1).

59.7% (3946/6615) of those who currently smoked tobacco were male, and the mean age was 63.9 years (SD 5.95). Most were of White ethnic backgrounds (75.3%; n=4982/6615) and lived in areas categorised within the two most deprived national socioeconomic quintiles (64.4%; n=4259/6615) (table 1).

**Table 1:** Characteristics of participants currently smoking tobacco who completed a baseline lung health check appointment between 8 April 2019 and 31 January 2020 (n=6615)

	Frequency (n)	Percentage (%)
Gender*		
Male	3946	59.7
Female	2669	40.3
Mean age†, (SD)	63.9 (5.95)	–
Age† groups		
55–59	1881	28.4
60–64	1842	27.8
65–69	1543	23.3
70–74	977	14.8
≥75 (maximum 78)	372	5.6
Ethnicity‡		
Asian	670	10.1
Black	451	6.8
Mixed	188	2.8
Other	324	4.9
White	4982	75.3
National IMD*		
Quintile 1 (most deprived)	2293	34.7
Quintile 2	1966	29.7
Quintile 3	1100	16.6
Quintile 4	896	13.5
Quintile 5 (least deprived)	293	4.4
Missing	67	1.0
Cigarette pack year history		
<20	786	11.9
20–39	2824	42.7
40–59	2101	31.8
60–79	446	6.7
80–99	170	2.6
≥100	150	2.3
Missing	138	2.1

* From primary care data.

† Age at time of baseline LHC appointment.

‡ Self-reported by participant at baseline LHC appointment.

IMDindex of multiple deprivation

### Acceptance of smoking cessation referral

6.2% (n=412/6615) of those who currently smoked tobacco were already in contact with an SSS and were excluded from further analysis. Of the 6203 remaining individuals, 33.7% (n=2090/6203) consented to a smoking cessation referral being made on their behalf. However, 42.7% (n=893/2090) of these individuals were residents in boroughs where local smoking cessation services were either not present or required individuals to self-refer (figure 1). These individuals were offered smoking cessation support cards, which were accepted by 90.5% (n=808/893) of individuals.

A further 18.1% (n=1124/6203) of individuals preferred to self-refer and 48.2% (n=2989/6203) declined smoking cessation support completely (figure 1).

Logistic regression analysis showed that males (adjusted OR (aOR) 1.16, 95% CI 1.01 to 1.34, p=0.037), younger individuals aged<70 years (55–59: aOR 1.70, 95% CI 1.22 to 2.35, p=0.002; 60–64: aOR 1.71, 95% CI 1.23 to 2.36, p=0.001 and 65–69: aOR 1.78, 95% CI 1.29 to 2.46, p<0.001) and those of ethnic minority backgrounds (Asian: aOR 1.31, 95% CI 1.02 to 1.68, p=0.033; Black: aOR 1.71, 95% CI 1.29 to 2.27, p<0.001 and Mixed: aOR 1.72, 95% CI 1.14 to 2.59, p=0.010) were more likely to consent to a smoking cessation referral being made on their behalf relative to females, individuals aged≥75 years and those of White ethnicity, respectively (table 2).

**Table 2:** Univariable and multivariable binary logistic regression analysis assessing characteristics of individuals who consented to a smoking cessation referral being made on their behalf (n=2090/6203)

	Frequency (n)	Percentage (%)	Univariable analysis		Multivariable analysis	
			Unadjusted OR, 95% CI	P value	aOR, 95% CI	P value
Gender						
Male	1248	59.7	0.98 (0.88 to 1.09)	0.753	**1.16(1.01to1.34)**	**0.037**
Female	842	40.3	1.00	–	1.00	–
Age groups						
55–59	647	31.0	**1.95(1.49to2.55)**	**<0.001**	**1.70(1.22to2.35)**	**0.002**
60–64	607	29.0	**1.80(1.38to2.36)**	**<0.001**	**1.71(1.23to2.36)**	**0.001**
65–69	502	24.0	**1.77(1.35to2.33)**	**<0.001**	**1.78(1.29to2.46)**	**<0.001**
70–74	254	12.2	1.27 (0.95 to 1.70)	0.103	1.20 (0.85 to 1.69)	0.306
≥75	80	3.8	1.00	–	1.00	–
Ethnicity						
Asian	225	10.8	**1.20(1.01to1.43)**	**0.038**	**1.31(1.02to1.68)**	**0.033**
Black	182	8.7	**1.60(1.30to1.95)**	**<0.001**	**1.71(1.29to2.27)**	**<0.001**
Mixed	70	3.3	**1.51(1.11to2.07)**	**0.009**	**1.72(1.14to2.59)**	**0.010**
Other	124	5.9	**1.43(1.13to1.80)**	**0.003**	1.26 (0.91 to 1.73)	0.165
White	1489	71.2	1.00	–	1.00	
National IMD						
Quintile 1 (most deprived)	653	31.2	**0.74(0.57to0.96)**	**0.023**	**0.65(0.47to0.89)**	**0.008**
Quintile 2	609	29.1	0.83 (0.64 to 1.07)	0.147	0.80 (0.58 to 1.10)	0.169
Quintile 3	356	17.0	0.88 (0.67 to 1.15)	0.339	0.95 (0.68 to 1.33)	0.766
Quintile 4	341	16.3	1.11 (0.84 to 1.46)	0.477	1.16 (0.82 to 1.62)	0.402
Quintile 5 (least deprived)	106	5.1	1.00	–	1.00	–
Missing	25	1.2	–	–	–	–
Cigarette pack years						
<20	242	11.6	1.21 (0.81 to 1.80)	0.361	1.63 (0.91 to 2.91)	0.102
20–39	933	44.6	1.33 (0.91 to 1.94)	0.142	1.24 (0.80 to 1.91)	0.338
40–59	650	31.1	1.25 (0.85 to 1.83)	0.260	1.17 (0.76 to 1.80)	0.482
60–79	121	5.8	1.03 (0.67 to 1.58)	0.902	0.97 (0.60 to 1.56)	0.887
80–99	50	2.4	1.20 (0.72 to 1.98)	0.485	1.06 (0.60 to 1.88)	0.838
≥100	39	1.9	1.00	–	1.00	–
Missing	55	2.6	–	–	–	–
Known COPD, chronic bronchitis or emphysema						
Yes	482	23.1	0.96 (0.85 to 1.09)	0.544	0.92 (0.79 to 1.07)	0.282
No	1608	76.9	1.00	–	1.00	–
Number of serious attempts to stop smoking in the last 12 months*						
0	760	36.4	1.00	–	1.00	
1–4	586	28.0	**1.75 (1.52 to2.00**)	**<0.001**	**1.65 (1.43 to1.90**)	**<0.001**
≥5	127	6.1	**1.49 (1.17 to1.89**)	**0.001**	**1.54 (1.20 to1.98**)	**<0.001**
Not available/do not know	617	29.5	–	–	–	–
Motivation to quit based on MTSS*						
High motivation to stop in a defined time frame	270	12.9	**2.16(1.80to2.59)**	**<0.001**	**1.92(1.58to2.34)**	**<0.001**
All other responses	1224	58.6	1.00	–	1.00	–
Not available	596	28.5	–	–	–	–
TTFC*						
<5 min	384	18.4	**1.69(1.38to2.07)**	**<0.001**	**2.07(1.65to2.59)**	**<0.001**
6–30 min	617	29.5	**1.39(1.13to1.64)**	**0.001**	**1.55(1.27to1.89)**	**<0.001**
31–60 min	283	13.5	**1.44(1.16to1.78)**	**<0.001**	**1.56(1.24to1.96)**	**<0.001**
>60 min	210	10.0	1.00	–	1.00	–
Not available	596	28.5	–	–	–	–

Values in bold are statistically significant.

* From participant questionnaire (see results for details of response options).

aORadjusted ORCOPDChronic obstructive pulmonary diseaseIMDindex of multiple deprivationMTSSmotivation to stop scaleTTFCtime to first cigarette

Conversely, individuals living in areas categorised within the most deprived national socioeconomic quintile (IMD 1: aOR 0.65, 95% CI 0.47 to 0.89, p=0.008) were less likely to consent to a smoking cessation referral compared with those living within the least deprived quintile (table 2).

Individuals reporting a high level of motivation to quit within the next 1 or 3 months (aOR 1.92, 95% CI 1.58 to 2.34, p<0.001) and those who had made one or more quit attempts in the past 12 months (1–4 attempts: aOR 1.65, 95% CI 1.43 to 1.90, p<0.001 and ≥5 attempts: aOR 1.54, 95% CI 1.20 to 1.98, p<0.001) were more likely to consent to a referral relative to those reporting low motivation to quit and those who had made no quit attempts, respectively. Similarly, TTFC of less than 60 minutes was associated with individuals being more likely to consent to a referral (<5 min: aOR 2.07, 95% CI 1.65 to 2.59, p<0.001; 6–30 min: aOR 1.55, 95% CI 1.27 to 1.89, p<0.001 and 31–60 min: aOR 1.56, 95% CI 1.24 to 1.96, p<0.001) compared with a TTFC of greater than 60 minutes (table 2).

No associations with consent to referral were identified across smoking pack year history and self-reported medical history of COPD, chronic bronchitis or emphysema.

### Smoking cessation referral outcomes

Outcome data were available for 742 referrals made to smoking cessation services by the study following an LHC appointment. The primary reason for missing outcome data was the decommissioning of services since the beginning of the study, meaning that the outcomes for individuals referred to these services were not available. In addition, some referrals could not be retrospectively identified by services due to the way these had been coded at the time of receipt.

Where outcome data were available, 47.3% (n=351/742) of individuals accepted the service when contacted. 16.7% (n=124/742) and 34.2% (n=254/742) declined and could not be contacted by the service, respectively (figure 1).

65.5% (n=230/351) of those who accepted the service were able to set a quit date. The overall 4-week quit rate among these individuals was 57.4% (n=132/230). This included 64 CO-validated and 68 self-reported quits. 40.0% (n=92/230) of individuals had not quit 4 weeks after setting a quit date and 2.6% (n=6/230) were lost to follow-up with no confirmation of 4-week smoking status (table 3).

**Table 3:** 4-week outcomes for individuals setting a quit date after attending an appointment with smoking cessation services (n=230)

	Total (n)	Total (% of individuals who set a quit date)	Total (% of individuals who accepted the service)	Total (% of referrals)
Total number of quit dates set	230	–	65.5%	31.0%
Total number of 4-weeks quits—overall	132	57.4%	37.6%	17.8%
*Total number of 4-week quits—CO validated*	*64*	*27.8%*	*18.2%*	*8.6%*
*Total number of 4-week quits—self-reported only*	*68*	*29.6%*	*19.4%*	*9.2%*
Total number of non-quits	92	40.0%	26.2%	12.4%
Total number lost to follow-up	6	2.6%	1.7%	0.8%

COcarbon monoxide

Among referrals with available outcome data, the 4-week quit rate was 17.8% (n=132/742), giving an overall ‘minimum’ quit rate of 6.3% (n=132/2090) among all individuals who consented to a referral.

## Discussion

To our knowledge, this is the first study in the UK to report service engagement outcomes following a VBA, ‘opt-out’ smoking cessation referral strategy in an LCS setting. Where outcome data were available, the overall 4-week quit rate among all individuals referred was 17.8%, giving a ‘minimum’ 4-week quit rate of 6.3% among all individuals who consented to a referral. Considering missing data and the fact that over 40% of these individuals could not be referred (eg, because they resided in boroughs with no local SSS), the ‘true’ 4-week quit rate in this cohort is likely to lie in between these two figures.

One-thirds (33.7%) of those not already in contact with SSS consented to a referral being made on their behalf. While this is comparable to the 36% uptake reported by NHS England’s Targeted Lung Health Check (TLHC) programme,^26^ it is significantly lower than the 88% and 83% uptake reported by YESS^15^ and the Cancer Care Ontario^27^ programme, respectively. Specialist smoking cessation support provided via these programmes was either co-located or built into baseline LDCT appointments for individuals eligible for screening, suggesting that the co-provision of expert smoking cessation support to individuals attending LCS appointments may be more effective in promoting referral acceptance. Potential reasons for this include convenience and specialist experience in engaging those with long-term tobacco dependence. In addition, this model enables support to be available straight after an LCS appointment, immediately harnessing the ‘teachable moment’ provided by screening.^28^ This significant difference in uptake suggests that the provision of on-site specialist smoking cessation support should be strongly advocated to maximise referral uptake in the context of large-scale, national LCS programmes.

An ‘opt-out’ smoking cessation referral strategy is currently recommended as a minimum standard of care by the national TLHC protocol. However, highlighting significant inadequacies in access to community smoking cessation services in the UK, over 40% of individuals who consented to a smoking cessation referral were resident in boroughs where local SSSs were either not available or required self-referral. As a result, SSS referrals could not be made on behalf of these individuals, representing a significant missed opportunity which must be addressed urgently in order to maximise the potential benefit of this referral strategy in an LCS setting. In addition, only 47% of individuals referred to SSS accepted an appointment with the service when contacted. These data require consideration when planning strategies for integrating smoking cessation interventions into a national LCS programme.

A lack of confidence, possibly related to a self-perceived lack of knowledge or expertise of the subject,^29^ is often a barrier to the delivery of smoking cessation interventions by healthcare professionals in an inpatient setting.^30^ While accredited VBA training was provided to all staff conducting LHC appointments, members of staff varied in terms of prior practical clinical experience. It is, therefore, possible that some staff members were more confident in giving VBA than others, also contributing to the relatively low referral acceptance rate. Nevertheless, it is worth noting that, while training healthcare professionals in the delivery of smoking cessation interventions has been shown to positively impact smoking prevalence and continuous abstinence among individuals who have quit,^31^ limited evidence from a community pharmacy setting suggests that the delivery of a standardised staff training programme has no impact on smoking cessation service uptake and retention.^32^ However, these data were based on an unselected cohort of individuals who smoked, rather than the population of high-risk individuals who would usually be invited to participate in LCS. Further research is required to help understand if these results are also applicable in an LCS setting.

Socioeconomic deprivation is thought to undermine smoking cessation efforts due to factors, including reduced social support and lower self-efficacy.^33^ Socioeconomic position also independently predicts the likelihood of long-term smoking abstinence.^34^ In keeping with these data, individuals in our cohort living in areas categorised within the most deprived national socioeconomic quintile were less likely to consent to a smoking cessation referral being made on their behalf, suggesting that more intensive and personalised approaches to offering support may be needed to help these individuals overcome their addiction.

A shorter TTFC was associated with a higher likelihood of smoking cessation referral acceptance, despite being recognised as a marker of greater tobacco dependence.^35^ This may reflect greater motivation to stop smoking in this cohort of individuals attending LCS,^36^ as well as recognition of the need for assistance to overcome their addiction. The LHC visit, therefore, provided an opportune moment for smoking cessation interventions to be offered to these individuals.

Nearly, two-thirds of those who accepted support from smoking cessation services following a referral from the study set a quit date, a practice which evidences the committed nature of the individual’s quit attempt and is associated with an increased likelihood of successful smoking cessation.^37^ The 57.4% quit rate among these individuals is similar to national data showing that 53.6% of all individuals seen in smoking cessation services between April and December 2022 successfully quit 4 weeks after setting a quit date.^38^

Including only referrals with available outcome data, the 4-week quit rate in our cohort was 18% among all individuals referred and 37% among those who accepted smoking cessation support. For comparison, outcomes reported by YESS show a 4-week quit rate of 17% among those accepting support.^39^ While it remains to be seen how our short-term quit rate translates into long-term abstinence, our figures compare favourably with the overall short-term quit rate of 11% seen in the UK Lung Cancer Screening (UKLS) trial in which individuals attending a baseline study visit were offered standard smoking cessation advice leaflets and given contact details for local smoking cessation services.^7^

Limitations of this study are recognised. Notably, this is an observational study, and no control arm was included in the analysis. This is particularly relevant in light of data from the Alberta screening cohort, which showed no difference in quit rates between individuals randomised to receive active smoking cessation counselling and controls.^40^ However, this study was limited to 345 individuals and may have been underpowered to detect a statistically significant difference in outcomes. Further research in a controlled, interventional setting is, therefore, required to help determine the most effective strategy for integrating smoking cessation interventions into LCS programmes. In addition, outcome data for several referrals made to SSS by the study were not available for analysis due to several factors, including the decommissioning of services since the beginning of the study, while it was also not possible to obtain equivalent data on individuals who were encouraged to self-refer to local SSS services or contact the Stop Smoking London Advice line. This potentially limits the impact of our findings. Finally, less than 50% of the successful 4-week quits reported in this analysis were CO verified, likely due to the impact of the SARS-CoV-2 pandemic on local SSS.

In conclusion, one-thirds of current smokers attending a baseline LHC to assess eligibility for LCS consented to a smoking cessation referral being made on their behalf. 4-week quit rates for individuals accepting smoking cessation support were higher than the quit rate among untreated smokers in the general population. Providing on-site specialist smoking cessation services is likely to boost uptake further and should be considered for future LCS programmes.

## supplementary material

10.1136/bmjresp-2024-002337 online supplemental file 1

## Data Availability

Data are available on reasonable request.
